# Supplementary material for: The mechanism of ribosomal recruitment during translation initiation on the Type 2 encephalomyocarditis virus IRES
Source: EMBO J. 2026 Mar 18;45(8):2666–93. doi: 10.1038/s44318-026-00735-x (PMC13084055; doi:10.1038/s44318-026-00735-x)
Supplement: Supplementary file 1 — Appendix [file 44318_2026_735_MOESM1_ESM.pdf]

APPENDIX for:

The mechanism of ribosomal recruitment during translation initiation on Type 2 IRESs

Sayan Bhattacharjee, Irina S. Abaeva, Zuben P. Brown, Yani Arhab, Hengameh Fallah, Christopher U. T. Hellen, Joachim Frank and Tatyana V. Pestova

Table of contents……………………………………………………………………………...……....1

Appendix Figure S1: Fourier Shell Correlation (FSC) curves for consensus and focused cryo-EM reconstructions ……………………………………………………………………………… ….……..2

Appendix Figure S2: Directed hydroxyl radical cleavage of the EMCV IRES in assembled 48S complexes from Fe(II) tethered to cysteines in eIF1A ……………………………………………………………......3

Appendix Figure S3: Protection of the EMCV IRES from RNAse T1 cleavage in 48S initiation complexes…...4

Appendix Fig. S4. The position of initiator tRNA in the EMCV IRES-containing and canonical 48S complexes..5

Appendix Figure S5: Consensus model of the apical region of domain I of type 2 IRESs ………….………...6

Appendix Table S1: Data collection statistics …………………………………………….……………7-8

Appendix Table S2: Search terms used to map visible IRES nucleotides in the cryo-EM structure of the 48S initiation complex assembled on the EMCV IRES to its nucleotide sequence…………..……..……………....9

Appendix Table S3: (related to Appendix Fig. S5): The apical region of domain I of picornavirus type 2 IRESs……………………………………………………………………………………………....10-11

Appendix Table S4: Primers used for toe-printing, directed hydroxyl radical cleavage and enzymatic foot-printing……………………………………………………...…………………………………………12

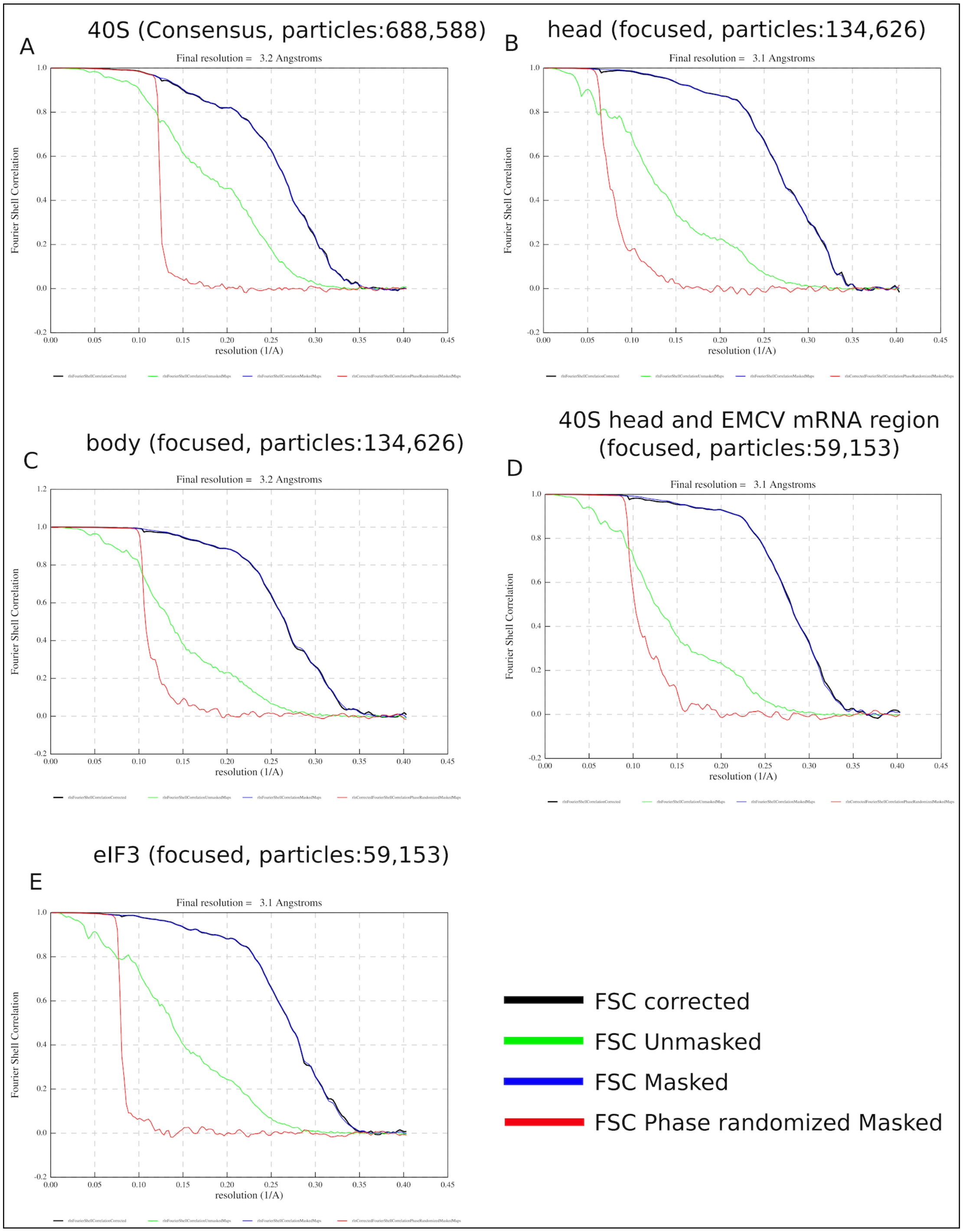

Appendix Fig. S1. Fourier Shell Correlation (FSC) curves for consensus and focused cryo-EM reconstructions.

(A) FSC curves for the global consensus reconstruction of the 40S subunit using 688,588 particles, yielding a final resolution of 3.2 Å. (B) Focused refinement on the 40S head from 134,626 particles achieved a resolution of 3.1 Å. (C) Focused refinement on the 40S body from the same particle subset reached a resolution of 3.2 Å. (D) Refinement focused on the 40S head and EMCV mRNA region from Class Ia (59,153 particles) resulted in a 3.1 Å resolution map. (E) Focused refinement on eIF3 from Class Ia (59,153 particles) also yielded a final resolution of 3.1 Å. FSC curves are shown for corrected (black), unmasked (green), masked (blue), and phase-randomized masked (red) data, with the 0.143 criterion used to determine final resolutions.

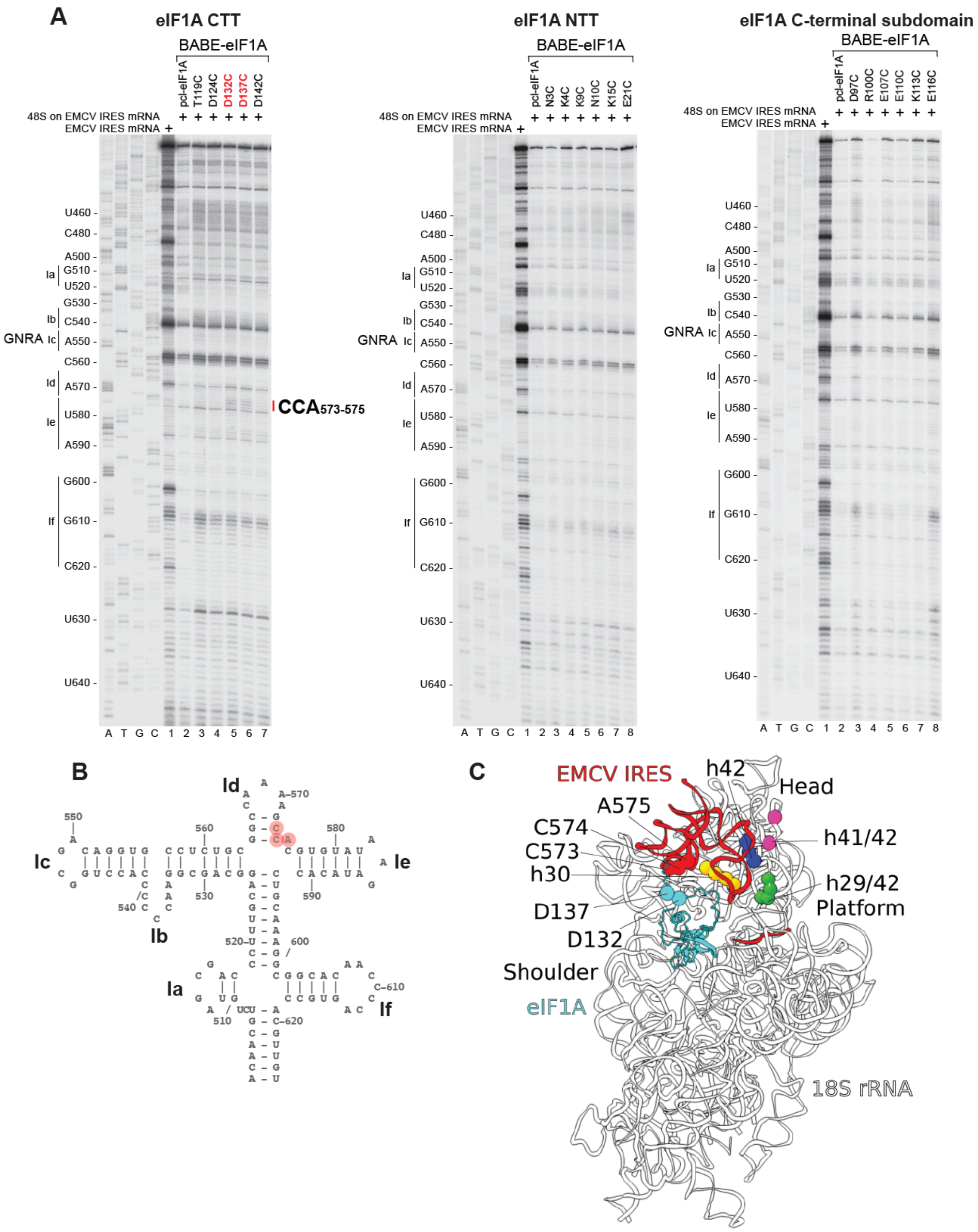

Appendix Fig. S2. Directed hydroxyl radical cleavage of the EMCV IRES in assembled 48S complexes from Fe(II) tethered to cysteines in eIF1A.

(A) Analysis of directed hydroxyl radical cleavage of the EMCV IRES in 48S complexes from cysteines in the C-terminal tail (CTT) (left panel), N-terminal tail (NTT) (middle panel) and C-terminal subdomain (right panel) of eIF1A. Sites of cleavage were mapped by primer extension inhibition. Positions of cleaved nucleotides are shown on the right. Lanes G, A, T, C depict EMCV sequence generated from the same primer. (B) Sites of directed hydroxyl radical cleavage in the EMCV IRES from D132C and D137C in eIF1A mapped onto the secondary structure of the apex of domain I. (C) Position of directed hydroxyl radical cleavage in the EMCV IRES mapped onto the cryo-EM structure of the 48S complex and positions of nucleotides in 18S rRNA that are cleaved from the same residues of eIF1A (D132C and D137C) in 43S preinitiation complexes (Yu et al., 2009).

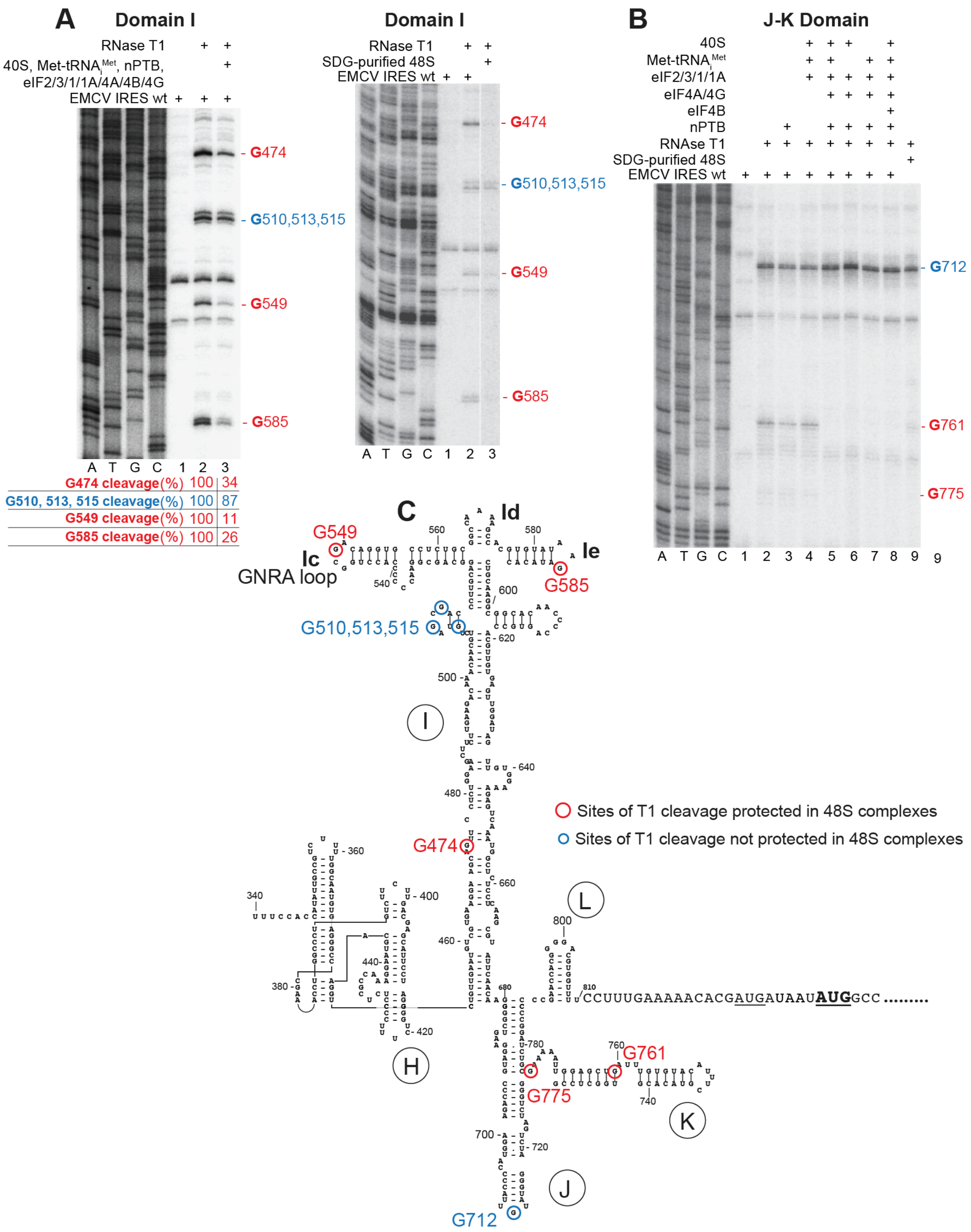

Appendix Fig. S3. Protection of the EMCV IRES from RNAse T1 cleavage in 48S initiation complexes.

(A) RNase T1 foot-printing of domain I in unpurified (left panel) and sucrose density gradient (SDG) purified (right panel) 48S complexes assembled on the EMCV IRES mRNA. The efficiency of T1 cleavage at the specific positions of the IRES was quantified taking the efficiency of cleavage of the individual IRES as 100%. Standard deviations (omitted for clarity) did not exceed 10%. (B) RNase T1 foot-printing of the JK domain in unpurified and SDG-purified 48S complexes assembled on the EMCV IRES mRNA. Sites of RNase T1 cleavage are marked on the right. Lanes C, T, A, and G depict EMCV sequence generated using the same primer. The division between lanes 2 and 3 (A, right panel) indicates that these two sets of lanes were derived from the same gel. (C) The EMCV IRES secondary structure model showing sites protected from the RNase T1 cleavage in 48S complexes.

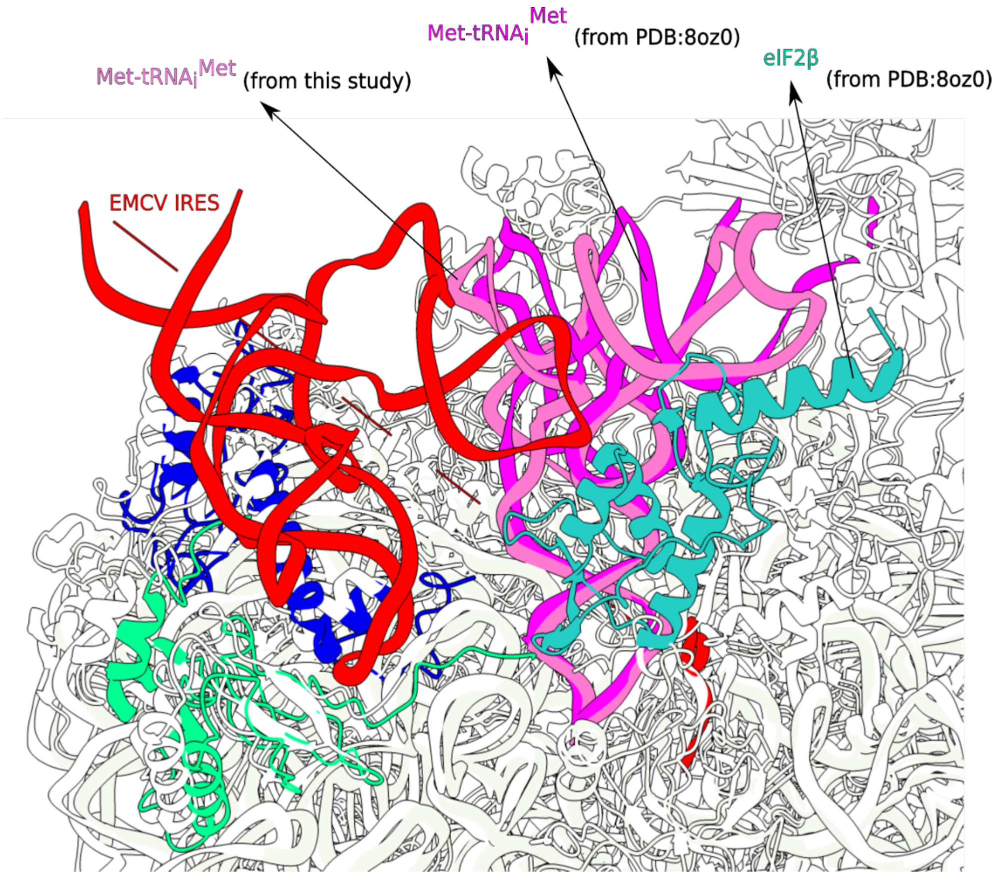

Appendix Fig. S4. The position of initiator tRNA in the EMCV IRES-containing and canonical 48S complexes.

Structural comparison reveals that the acceptor arm of the P-site-bound tRNA undergoes a displacement from its position associated with eIF2β in canonical 48S complexes (PDB id: 8oz0) to a new position where it interacts directly with the EMCV IRES.

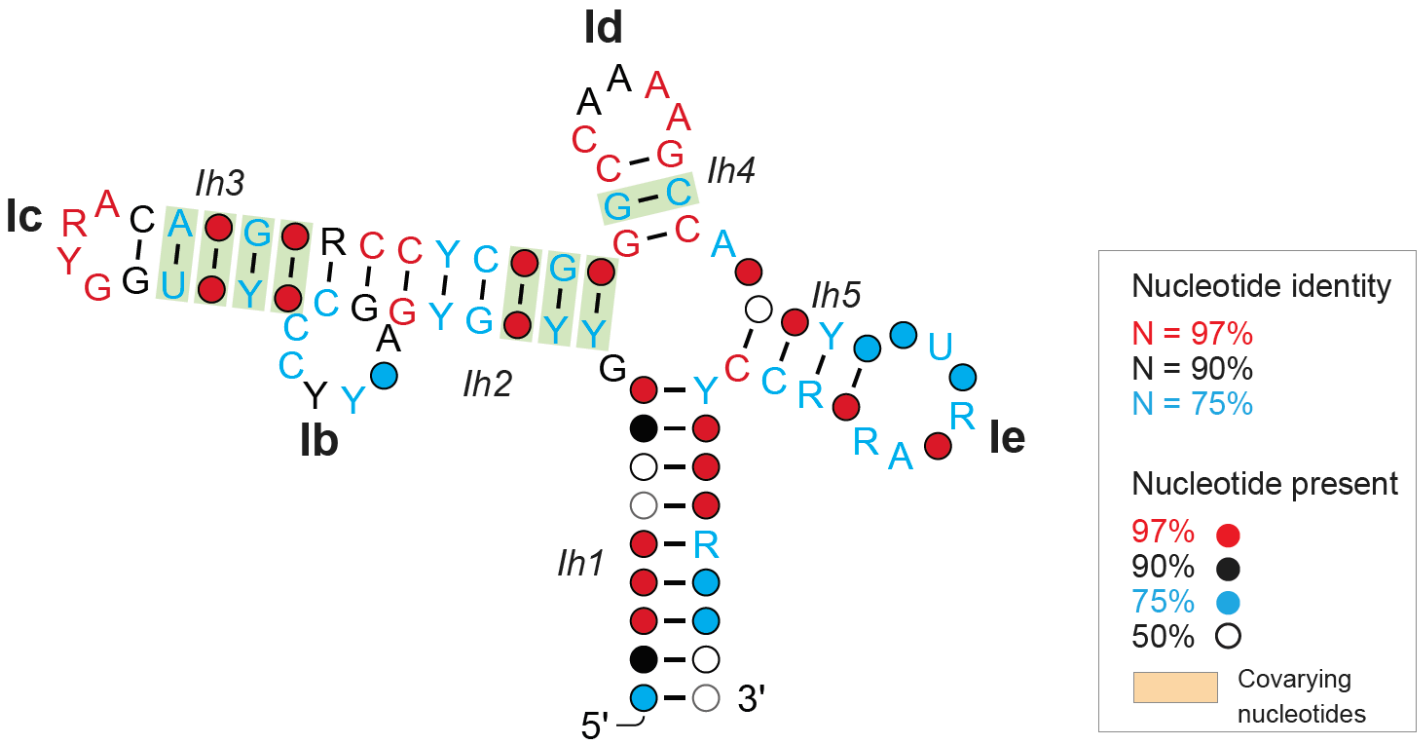

Appendix Fig. S5. Consensus model of the apical region of domain I of type 2 IRESs.
The covariance model was derived using R-Scape and CaCoFold (Rivas et al., 2017; Rivas, 2020) using 89 curated picornavirus sequences (see Appendix Table S3) and summarized as a secondary structure with significantly covarying positions indicated by green highlighting. Y = U or C, R = A or G. The model is annotated to indicate structural elements.

Appendix Table S1. Data collection statistics.

Parameter	Value
Microscope	FEI Titan Krios
Voltage	300 kV
Detector	Gatan K3 Summit
Energy Filter	Gatan BioQuantum, 20 eV slit width
Magnification	105,000×
Pixel size	0.83 Å
Electron dose	~60 $e^{-}/Å^2$ (total)
Exposure time	2.5 s (fractionated into 50 frames)
Defocus range	–0.8 to –2.5 μm
Number of micrographs collected	15,446
Number of particles extracted	835,875
Number of particles after 2D classification	688,588
Symmetry imposed	C1
Final map resolution (FSC 0.143)	3.1 Å
Map sharpening B-factor	–85 $Å^2$
Software used	cryoSPARC, RELION, Phenix, COOT
Model building & refinement	Phenix, Coot

Model resolution (FSC 0.5, masked)	3.3 Å
MolProbity score	1.75
Clashscore	6.8
Ramachandran favored (%)	96.2
Ramachandran outliers (%)	0.1
Rotamer outliers (%)	0.5
CC (map-to-model)	0.84

Appendix Table S2. Search terms used to map visible IRES nucleotides in the cryoEM structure of the 48S initiation complex assembled on the EMCV IRES to its nucleotide sequence.

Direction	Search terms	Hits	Possible
5' to 3'	_RNRR	39	2
5' to 3'	_RYRR	23	1
5' to 3'	_RYRRNNNNNNNNYY	9	1
5' to 3'	_RYRRYNNNNNNNYY	6	1
5' to 3'	YRYRRNNNNNNNNYY	5	1
5' to 3'	YRYRRYNNNNNNNYY	3	1
5' to 3'	YRYRRNNNNRNNYY	1	1
3' to 5'	RRNR_	42	2
3' to 5'	RRYR_	18	1
3' to 5'	YYNNNNNNNNRRYR_	7	1
3' to 5'	YYNNNNNNNNRRYRY	3	1
3' to 5'	YYNNNNNNNYRRYRY	0	0
3' to 5'	YYNNRNNNNRRYRY	0	0

Appendix Table S3 (related to Appendix Fig. S5). The apical region of domain I of picornavirus type 2 IRESs

Genus	Virus	Acc. No.	Nts.
Ailurivirus	Ailurivirus D isolate QlGpf124Aim01-12	MZ357174.1	886-968
Aphthovirus	Foot-and-mouth disease virus A isolate abrazil iso67	AY593788.1	779-857
	Foot-and-mouth disease virus A isolate Hafizabad/QOL-UVAS-Pak/2005	KY446902.1	769-847
	Foot-and-mouth disease virus A isolate Egypt/El Minia/36/2016	ON168597.1	58-135
	Foot-and-mouth disease virus A isolate a3mecklenburg iso81	AY593776.1	737-815
	Foot-and-mouth disease virus SAT 2 isolate BOT-BUFF/107/72	MH053331.1	735-811
	Foot-and-mouth disease virus A isolate MCH/1557/2001	OR338615.1	747-824
	Foot-and-mouth disease virus Asia1/PAK/ICT/220-4/2012_pro	OM471639.1	774-852
	Foot-and-mouth disease virus (FMDV) strain C, isolate c-s8c1	AJ133357.1	726-804
	Foot-and-mouth disease virus A isolate A/ALG/3/2017	MG923580.1	244-321
	Foot-and-mouth disease virus O isolate O1/Manisa/TUR/69	KY825719.1	199-277
Bopivirus	Bopivirus sp. strain goat/AGK16/2020-HUN	MW298058.1	236-315
	Bopivirus sp. strain deer/VIC82-2020/AUS	MZ436972.1	301-378
Cardiovirus	Boone cardiovirus 1 isolate BCV-1	NC_038305.1	1114-1194
	Cardiovirus A isolate 22084x5-1820	MZ544194.1	252-334
	Cardiovirus B strain rat08/rCaB/HUN	MN116646.1	753-834
	Cardiovirus B isolate YNMIX-YNSC213	PQ678021.1	650-732
	Cardiovirus F1 isolate RtMruf-PicoV/JL2014-1	NC_075977.1	499-580
	Vole cardiovirus isolate 14657	ON584156.1	576-658
	Encephalomyocarditis virus	NC_001479.1	518-600
	Encephalomyocarditis (EMC) virus EMC-D variant	M22458.1	513-595
	Mengo virus isolate Rz-pMwt	DQ294633.1	437-519
	Mengo virus strain AnrB-3741	KU955338.1	230-312
	Rat theilovirus 1 strain RTV-1	EU542581.1	742-823
	Cardiovirus B strain Ruian-Rn93-3	MF352411.1	179-260
	Cardiovirus B3 isolate 1	MF172923.1	757-837
	Genet fecal theilovirus isolate S15	KF823815.1	1114-1194
	Theiler's murine encephalomyelitis virus GDVII	X56019.1	751-833
	Marmot cardiovirus strain HHMCDV	MZ382838.1	740-821
	Cardiovirus C3 strain Wencheng-Rn416	MF352424.1	1000-1081
	Saffold virus strain Can112051-06	JF813004.1	738-819
	Saffold virus strain Penang	HQ162476.1	755-836
	Saffold virus: isolate: Pak-2491	AB747249.1	734-815
	Saffold virus strain Nijmegen2008	FN999911.1	735-816
Cosavirus	Cosavirus A isolate MR96-15-1/GER/2015	MT094345.1	245-327
	Cosavirus A strain AM326/BRA-AM/2017	MT023104.1	850-932
	Cosavirus B isolate A21_AFP15_NGR_2020-B	PP386518.1	260-342
	Cosavirus E isolate A28_AFP12_NGR_2020-E	PP386515.1	640-720
	Human cosavirus isolate SEWAGE/NL/1999-046-1	KJ437094.1	223-305
	Human cosavirus A19 strain PK6187	JN867759.1	45-125
Erbovirus	Equine rhinitis A virus strain PERV-1	DQ272578.1	622-699
	Equine rhinitis A virus strain Plowright	DQ272127.1	278-355
	Erbovirus A strain 303	KX260138	547-623
	Erbovirus A strain 396	KX260139.1	550-626
	Erbovirus A strain 421	KX260140.1	547-627
	Equine rhinitis B virus 1	NC_003983	543-621
	Equine rhinitis B virus 2 strain 1228	KX260141.1	555-631
	Equine rhinovirus 3 strain P313/75	AF361253	555-631
	Crocidura shantungensis picorna-like virus 5 isolate picorna_8	PP272660.1	343-423
	Picornavirales sp. isolate 193-k141_280731	MZ678985.1	440-519
	Wufeng shrew picornavirus 3 isolate WF_Cr.attenuata_picorna_2	OQ716066.1	7--781
Hunnivirus	Hunnivirus A9 isolate RtRrs-PicoV/YN2014	KY432925.1	344-422
	Hunnivirus A isolate JM_Ap.agrarius_picorna_1	OQ715979.1	369-449
	Hunnivirus A7 isolate 05VZ-75-RAT099	KT944214.1	271-351
	Hunnivirus A isolate LQ_Ra.tanezumi_picorna_1	OQ715984.1	366-446
	Pangolin hunnivirus isolate ZJ-MO7	OM451179.1	404-483
	Bovine hunnivirus strain BoHuV-WZ-202	OQ790152.1	252-330
	Ovine hungarovirus OHUV1/2009/HUN	HM153767.3	389-469
	Porcupine hunnivirus isolate FJ-F1	OM451178.1	345-425
Mischivirus	Mischivirus sp. isolate MSWZC17/6	OR867092.1	1085-1164
	Cabezo Gordo bat-associated mischivirus	PP654855.1	1106-1185

	Miniopterus schreibersii picornavirus 1	NC_034381.1	1093-1172
	African bat icavirus A isolate PREDICT-06105	KP100644.1	835-912
	Pteropus rufus mischivirus isolate AMB150	OQ818316.1	829-910
	Miniopterus bat picornavirus isolate 2A/Kenya/BAT0738/2015	PP711935.1	1107-1186
	Canine picornavirus isolate A128thr polyprotein (QKD15_gp1)	NC_075428.1	573-651
**Mosavirus**	Mosavirus sp. isolate YSS01	MW826550.1	336-409
	Mosavirus A2 strain SZAL6-MoV/2011/HUN	NC_023987.1	352-425
**Parechovirus**	Sebokele virus 1	NC_021482.1	418-495
	Ljungan virus strain 87-012G	EF202833.1	422-501
	Ljunganvirus 5	LC133331.1	409-487
	Bovine parechovirus cow/2018/4	BR001751.1	376-458
	Bovine parechovirus Bo_Par/Den1/2021/JPN	LC650808.1	376-458
	Parechovirus C isolate 22057x67-9	MZ544294.1	117-197
	Parechovirus E1 isolate falcon/HA18-080/2014/HUN	KY645497.1	444-522
**Rabovirus**	Rabovirus A strain Wencheng-Rt38-1	MF352417.1	408-486
**Rosavirus**	Rosavirus M-7	NC_038880.1	163-247
	Rosavirus B isolate YY4	PQ045668.1	51-134
	Rosavirus B isolate RVB/YY86	OM492423.1	151-232
	Rosavirus B isolate RVB/YY106	OM492427.1	201-282
	Rosavirus B strain rat08/rRoB/HUN	MN116648.1	235-316
	Rosavirus B isolate RVB/SZ59	OM492432.1	139-221
	Rosavirus C strain RASM14A	KX783433.1	373-455
	Rosavirus C strain NFSM6F	KX783428.1	207-291
**Sapelovirus**	Coypu sapelovirus 1 isolate GX-F1	OM451188.1	420-495
	Coypu sapelovirus 2 isolate HuN-A2	OM451191.1	450-526
**Unclassified**	Picornavirales sp. isolate 193-k141_280731	MZ678985.1	440-519
	Riboviria sp. isolate flycatcher172_contig_428	OQ424099.1	6689-6608
	Picornaviridae sp. isolate xizangnaqu19-7207	OR367578.1	443-521
	Picornaviridae sp. isolate YSN02	MW826507.1	263-335

Names, accession numbers and inclusive nucleotide numbers of sequences from the named picornaviruses, belonging to genera as indicated, that belong to the apical region of domain I. Sequences were analyzed using R-scape; Cacofold was then used to generate a consensus secondary structure (Appendix Fig. S10).

Appendix Table S4. Primers used for toe-printing, directed hydroxyl radical cleavage and enzymatic foot-printing.

Primer sequence (5'-3')	Complementary position on the EMCV mRNA
CGGTATTGTAGAGCAG	nt. 901-916
GCAGGTAAAATCCATTACGG	nt. 914-933
GCCCCTTGTTGAATACGCTT	nt. 665-684
GCAAGTCTCTTGTTCCATGG	nt. 844-863
