# Supplementary figures and images for: The mechanism of ribosomal recruitment during translation initiation on the Type 2 encephalomyocarditis virus IRES
Source: EMBO J. 2026 Mar 18;45(8):2666–93. doi: 10.1038/s44318-026-00735-x (PMC13084055; doi:10.1038/s44318-026-00735-x)

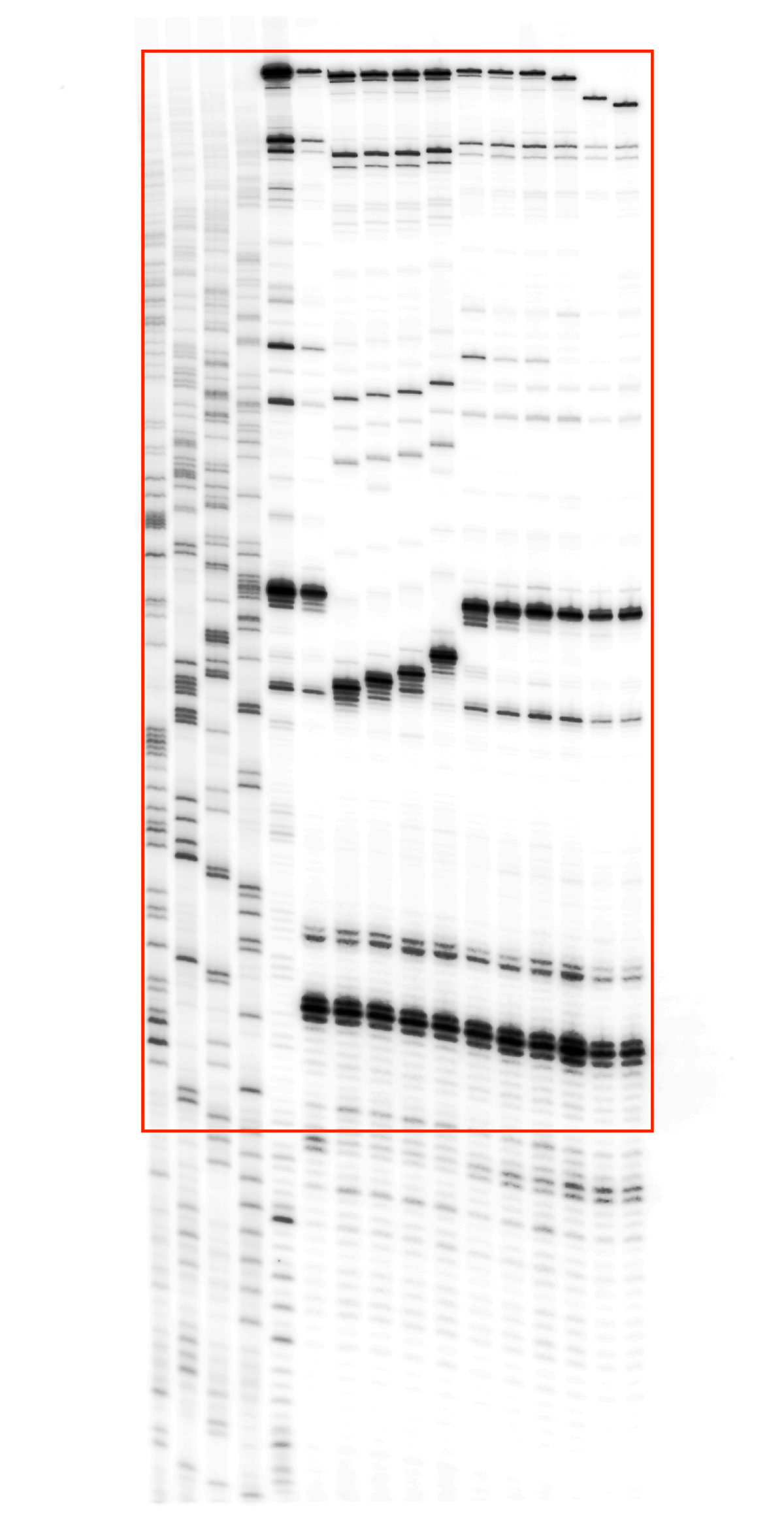

Supplement: Supplementary file 5 — Source data Fig. 7 [file 44318_2026_735_MOESM5_ESM.zip › 7B.tif]

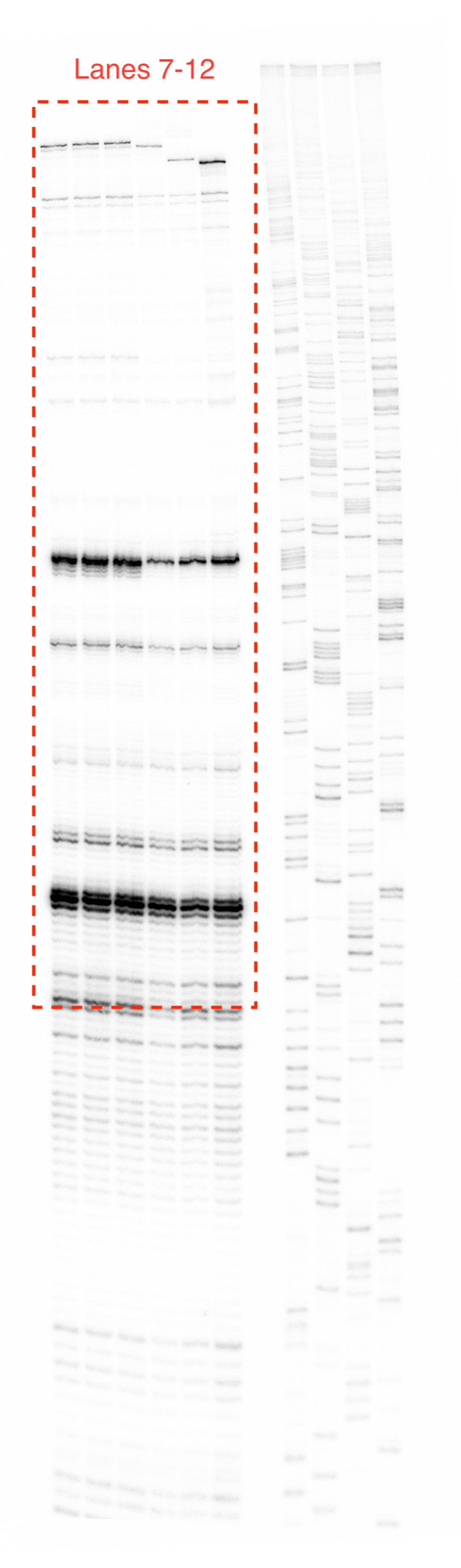

Supplement: Supplementary file 5 — Source data Fig. 7 [file 44318_2026_735_MOESM5_ESM.zip › 7B lanes 7-12 replica 3.tif]

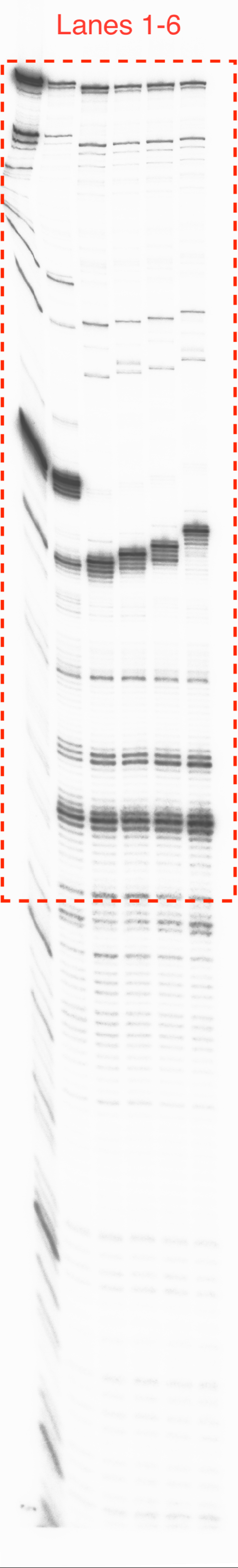

Supplement: Supplementary file 5 — Source data Fig. 7 [file 44318_2026_735_MOESM5_ESM.zip › 7B lanes 1-6 replica 2.tif]

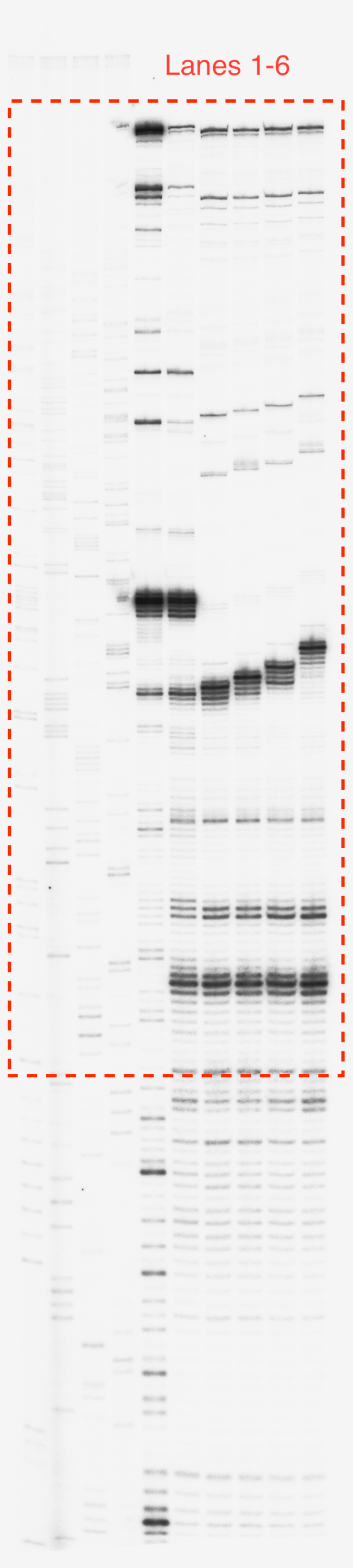

Supplement: Supplementary file 5 — Source data Fig. 7 [file 44318_2026_735_MOESM5_ESM.zip › 7B lanes 1-6 replica 1.tif]
