# Supplementary material for: The mechanism of ribosomal recruitment during translation initiation on the Type 2 encephalomyocarditis virus IRES
Source: EMBO J. 2026 Mar 18;45(8):2666–93. doi: 10.1038/s44318-026-00735-x (PMC13084055; doi:10.1038/s44318-026-00735-x)
Supplement: Supplementary file 7 — Expanded View Figures [file 44318_2026_735_MOESM7_ESM.pdf]

Expanded View Figures

Figure EV1. Secondary structure of the EMCV IRES.

The EMCV IRES secondary structure model featuring individual domains, the region corresponding to cryo-EM density (red), and specific binding of eIF4A/4G to the JK domain.

© The Author(s)

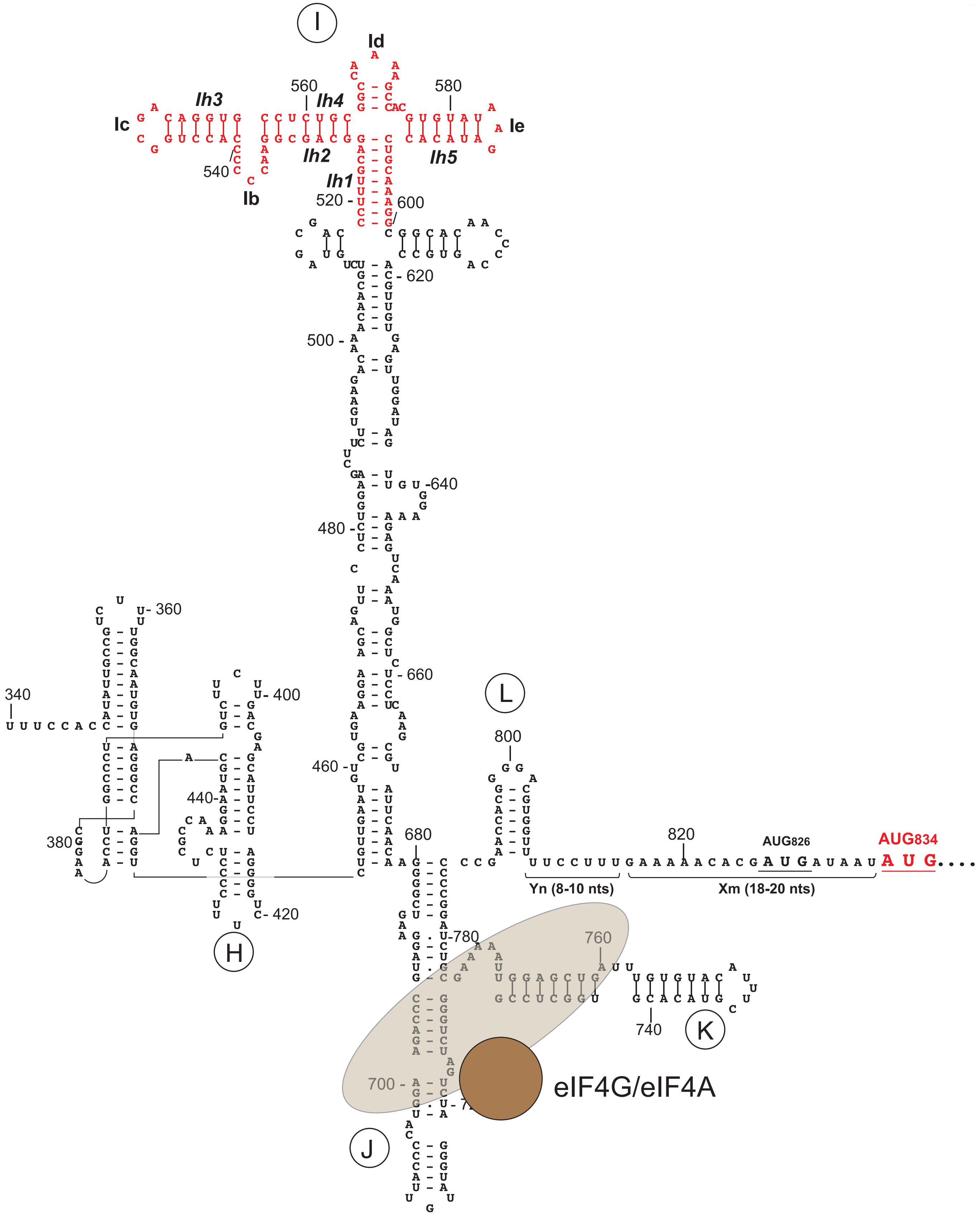
I
Id
Ic
Ie
Ib
Ih1
Ih2
Ih3
Ih4
Ih5
340
360
380
400
420
440
460
480
500
520
540
560
580
600
620
640
660
680
700
740
760
780
800
820
H
J
K
L
eIF4G/eIF4A
Yn (8-10 nts)
Xm (18-20 nts)
AUG826
AUG834
U U C C U U U G A A A A A C A C G A U G A U A A U A U G. . . .

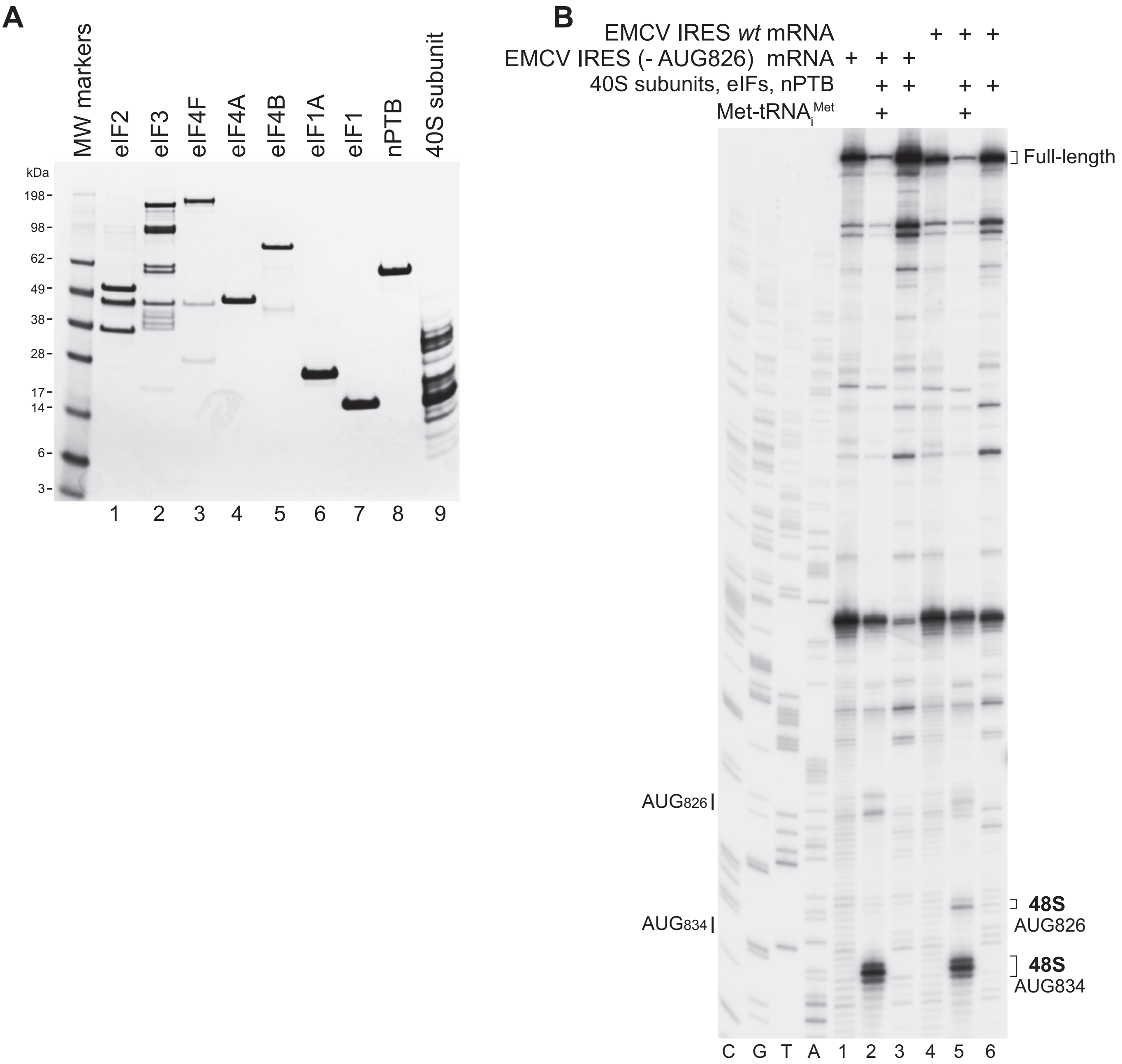

Figure EV2. 48S complex formation on the *wt* EMCV IRES and the IRES with mutated AUG_{826}.

(**A**) Purified initiation factors, nPTB and 40S subunits, analyzed by SDS-PAGE followed by SimplyBlue staining. (**B**) Toe-printing analysis of 48S complex formation on *wt* EMCV IRES mRNA and IRES mRNA with mutated AUG_{826} in the presence of 40S subunits, initiator Met-tRNA$_i^{Met}$, nPTB and indicated initiation factors. Lanes C, T, A, and G depict wt EMCV sequence generated using the same primer. The positions of initiation codons are indicated on the left and of assembled 48S complexes on the right.

 © The Author(s)

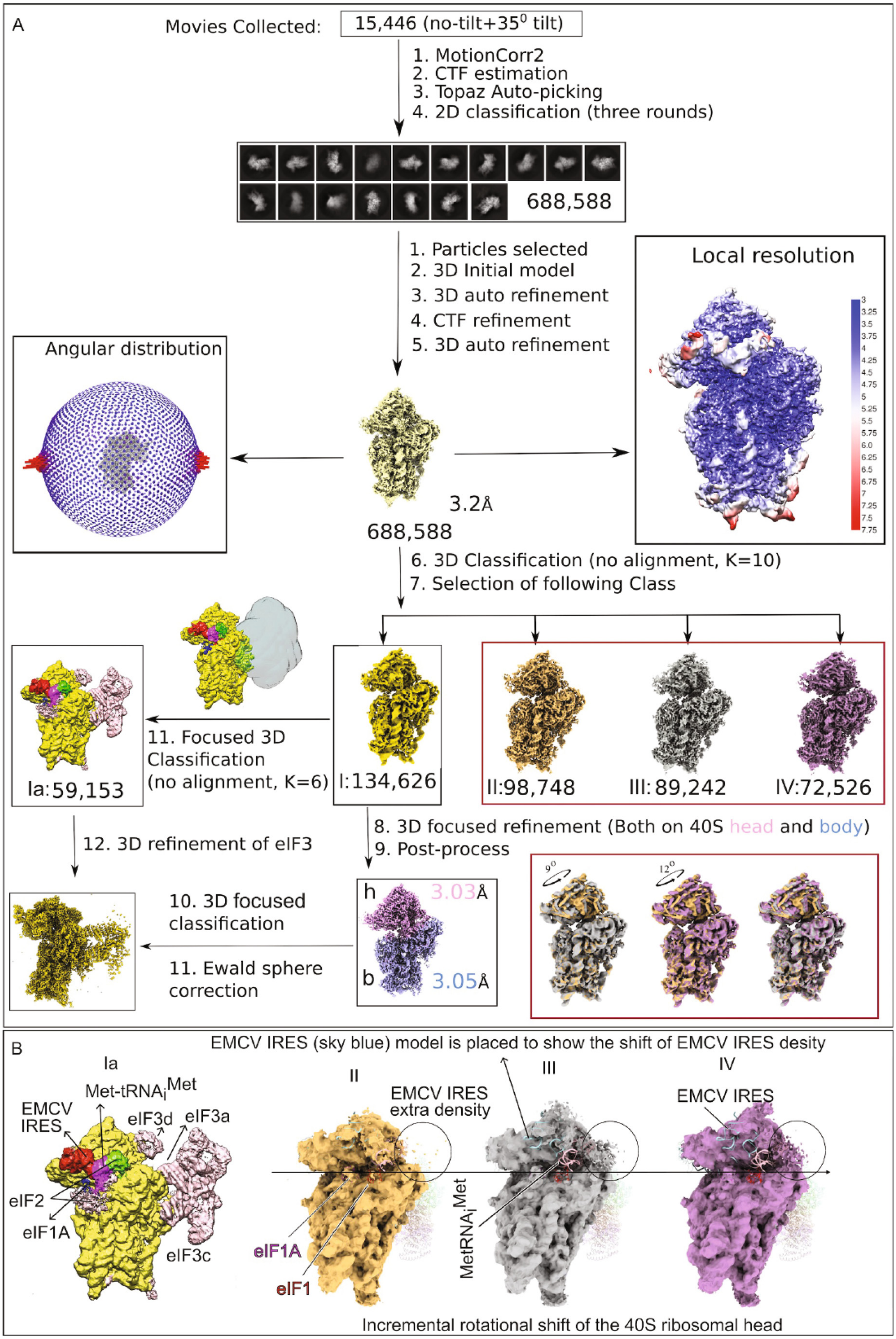
A
Movies Collected:
15,446 (no-tilt+35⁰ tilt)
1. MotionCorr2
2. CTF estimation
3. Topaz Auto-picking
4. 2D classification (three rounds)
688,588
1. Particles selected
2. 3D Initial model
3. 3D auto refinement
4. CTF refinement
5. 3D auto refinement
Local resolution
Angular distribution
3.2Å
688,588
6. 3D Classification (no alignment, K=10)
7. Selection of following Class
11. Focused 3D Classification (no alignment, K=6)
Ia:59,153
I:134,626
II:98,748
III: 89,242
IV:72,526
8. 3D focused refinement (Both on 40S head and body)
9. Post-process
12. 3D refinement of eIF3
10. 3D focused classification
11. Ewald sphere correction
h 3.03Å
b 3.05Å
B
EMCV IRES (sky blue) model is placed to show the shift of EMCV IRES desity
Ia
II
III
IV
Met-tRNAiMet
EMCV IRES
eIF3d
eIF3a
eIF2
eIF1A
eIF3c
EMCV IRES extra density
EMCV IRES
MetRNAiMet
eIF1A
eIF1
Incremental rotational shift of the 40S ribosomal head

◀ **Figure EV3. Cryo-EM data processing workflow and structural characterization of EMCV IRES 48S initiation complexes.**

(**A**) Data processing pipeline for structure determination of EMCV IRES 48S initiation complexes. (**B**) Class Ia (left): 48S initiation complex showing Met-tRNA$_i^{Met}$, eIF2, eIF1A, eIF3 (eIF3a/c/d subunits are indicated), and EMCV IRES density bound at the 40S subunit head. Classes II–IV: A series of reconstructions capturing progressive 40S head rotation, accompanied by repositioning of the EMCV IRES. Fragmented extra density attributed to the IRES is highlighted (circled), and a sky-blue model of the EMCV IRES is overlaid to illustrate its positional shift. Gaussian filtering reveals that this density remains connected to the IRES across all states. The horizontal arrow from left to right denotes the direction of progressive rotational movement of the 40S head, representing a continuum of structural intermediates.

© The Author(s)

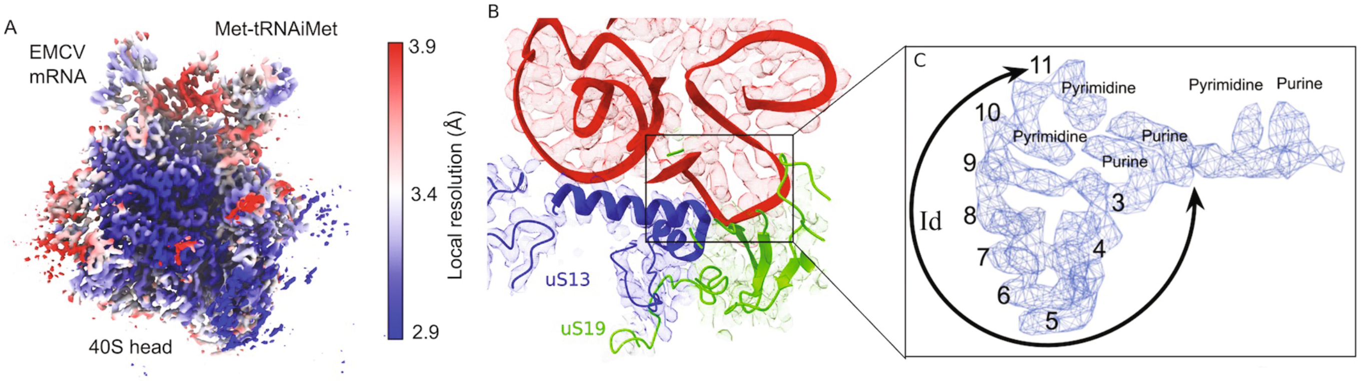

Figure EV4. Local resolution estimation and assignment of nucleotides of EMCV mRNA.

(**A**) The local resolution estimation of the 40S head part along with the EMCV IRES and Met-tRNA$_i^{Met}$. (**B**, **C**) The zoomed view of the Id subdomain of the EMCV IRES (in mesh) showing the loop formation through base pairing among RNA bases. The models fitted within densities of the EMCV IRES (red), uS19 (green), and uS13 (blue) are showing the relative position of Id on the 40S head.

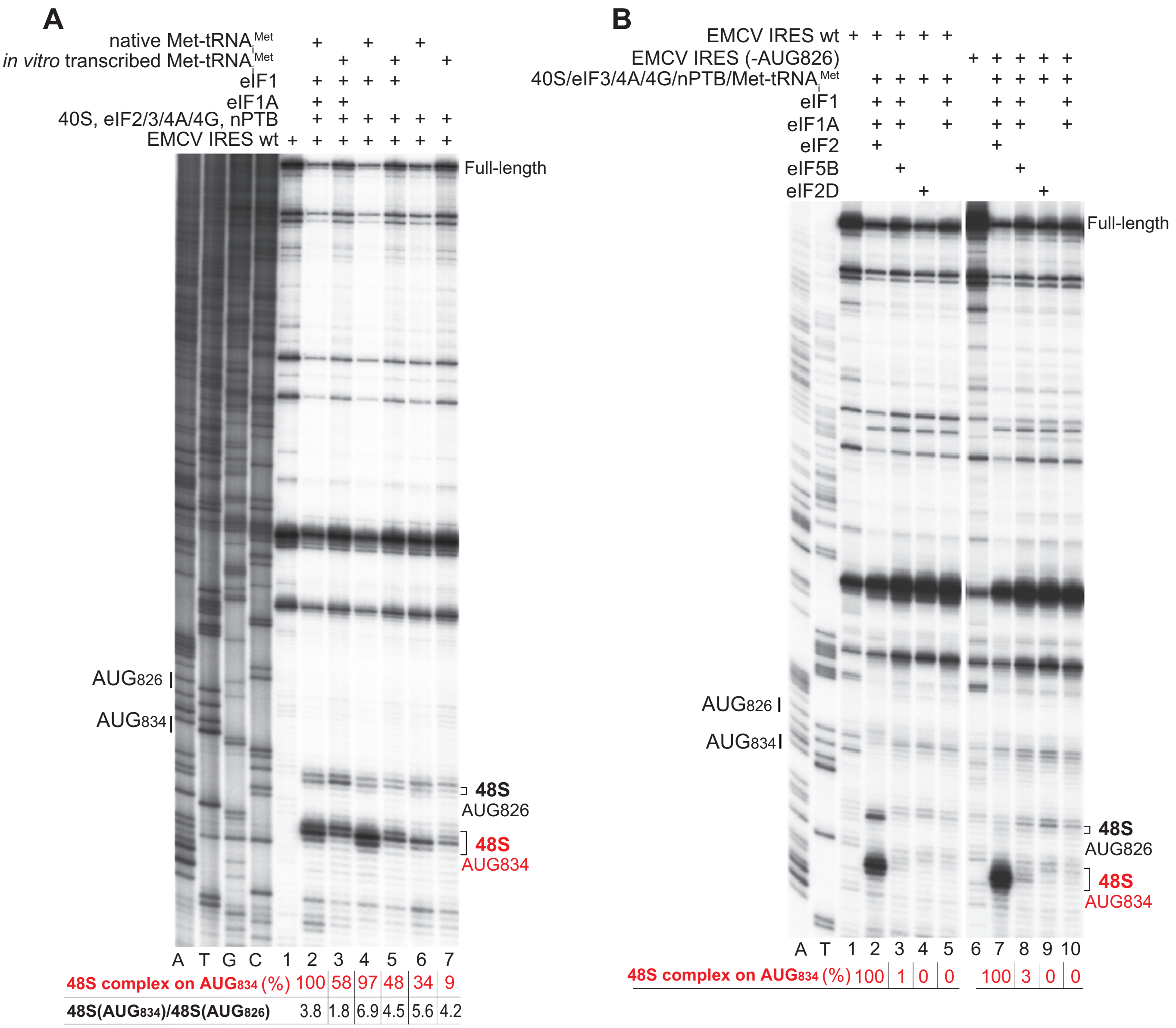

Figure EV5. Influence of the nature of initiator tRNA and substitution of eIF2 by eIF5B or eIF2D on 48S complex formation on the EMCV IRES.

(**A**) Toe-printing analysis of 48S complex formation on wt EMCV IRES mRNA in the presence of either native or in vitro transcribed Met-tRNA$_i^{Met}$, 40S subunits, nPTB and indicated initiation factors. The efficiency of 48S complex formation on AUG$_{834}$ (red numbers) was quantified on the basis of three experiments with the efficiency of complex formation in the presence of eIF1, eIF1A and native Met-tRNA$_i^{Met}$ defined as 100%. Standard deviations (omitted for clarity) did not exceed 8%. Black numbers represent relative efficiencies of 48S complex formation on AUG$_{834}$ and AUG$_{826}$ in each condition. (**B**) Toe-printing analysis of 48S complex formation on wt EMCV IRES mRNA and mRNA with mutated AUG$_{826}$ in the presence of 40S subunits, initiator tRNA, nPTB and indicated initiation factors. The efficiency of 48S complex formation on AUG$_{834}$ (red numbers) was quantified taking the efficiency of complex formation in the presence of eIF2 defined as 100%. Standard deviations (omitted for clarity) did not exceed 15%. (**A**, **B**) Lanes C, T, A, and G depict wt EMCV sequence generated using the same primer. The positions of initiation codons are indicated on the left and of assembled 48S complexes on the right. The division between lanes 5 and 6 (**B**) indicates that these two sets of lanes were derived from the same gel.

 © The Author(s)
